# CmDOF18 positively regulates salinity tolerance in *Chrysanthemum morifolium* by activating the oxidoreductase system

**DOI:** 10.1186/s12870-024-04914-y

**Published:** 2024-04-01

**Authors:** Peiling Li, Tingting Fang, Xinran Chong, Juanjuan Chen, Jianhua Yue, Zhiyong Wang

**Affiliations:** 1 College of Horticulture, Xinyang Agriculture and Forestry University, Xinyang, 464000 China; 2 Jiangsu Key Laboratory for the Research and Utilization of Plant Resources, Institute of Botany, Jiangsu Province and Chinese Academy of Sciences, Nanjing Botanical Garden Mem. Sun Yat-Sen, Nanjing, 210000 China

**Keywords:** *Chrysanthemum morifolium*, Salinity stress, DOF transcription factor

## Abstract

**Background:**

Chrysanthemum, one of the four major cut flowers all over the world, is very sensitive to salinity during cultivation. DNA binding with one finger (DOF) transcription factors play important roles in biological processes in plants. The response mechanism of *CmDOF18* from chrysanthemum to salt stress remains unclear.

**Results:**

In this study, *CmDOF18* was cloned from *Chrysanthemum morifolium*, and its expression was induced by salinity stress. The gene encodes a 291-amino acid protein with a typical DOF domain. CmDOF18 was localized to the nucleus in onion epidermal cells and showed transcriptional activation in yeast. *CmDOF18* transgenic plants were generated to identify the role of this gene in resistance to salinity treatment. Chrysanthemum plants overexpressing *CmDOF18* were more resistant to salinity stress than wild-type plants. Under salinity stress, the malondialdehyde content and leaf electrolyte conductivity in *CmDOF18*-overexpressing transgenic plants were lower than those in wild-type plants, while the proline content, chlorophyll content, superoxide dismutase activity and peroxidase activity were higher than those in wild-type plants. The opposite findings were observed in gene-silenced plants compared with wild-type plants. The gene expression levels of oxidoreductase increased in *CmDOF18*-overexpressing transgenic plants but decreased in *CmDOF18*-SRDX gene-silenced transgenic plants.

**Conclusion:**

In summary, we analyzed the function of *CmDOF18* from chrysanthemum, which may regulate salinity stress in plants, possibly due to its role in the regulation of oxidoreductase.

**Supplementary Information:**

The online version contains supplementary material available at 10.1186/s12870-024-04914-y.

## Background

Transcription factors (TFs) activate or inhibit target gene transcription by directly binding to cis-regulatory elements of promoters acting as gene expression regulators [1, 2]. The DNA binding with one finger (DOF) family is a classic protein in the Cys2-His2 zinc finger superfamily of TFs [3]. The DOF TFs contain a single conserved zinc finger motif named the DOF domain with a Cys2-His2 zinc finger containing 50–52 amino acid residues that binds to a specific element with 5’-AAAG-3’ sequences [4, 5]. In recent years, numerous members of the DOF TF family have been reported in a diverse variety of plants [6–13]. There are 36, 30 and 78 members of the DOF family in Arabidopsis (*Arabidopsis thaliana*), rice (*Oryza sativa*) and soybean (*Glycine max*), respectively [14, 15].

DOF TFs are involved in growth [16–18], development [19–21], abiotic stress [10, 22], hormone signaling [23, 24], and light signal transduction [16] in various plants. For example, in *A. thaliana*, the RGL2-DOF6 complex, in which *AtDOF6* interacts with RGL2, promotes seed dormancy by binding to the downstream *AtGATA12* promoter [25]. The expression of the *BnCDF1* gene is induced in response to low temperatures, and Arabidopsis plants overexpressing *BnCDF1* exhibit increased freezing tolerance, and delayed flowering time because of modulation of the expression patterns of CO and FT flowering time control genes [18]. *FcDof4* and *FcDof16* trigger flavonoid C-glycosyltransferase (*FhCGT*) expression by specifically binding to the gene promoters to promote flavonoid synthesis in kumquat fruit [26]. Twenty-five *ChDof* genes have been identified in the *Cerasus humilis* genome, and qRT-PCR analysis of the expression patterns of the genes in fruit during storage have suggested that these genes might play important roles in fruit storage [13]. Numerous DOF genes have been identified as being involved in different physiological processes in plants [6, 9, 11, 15, 27–29].

Chrysanthemum (*Chrysanthemum morifolium*) is one of the most popular cut flowers worldwise and is grown widely for its ornamental and medicinal value. Chrysanthemum plants are susceptible to salinity stress, which causes extensive leaf chlorosis, retards growth, and in some cases even kills the plant [30, 31]. Transcriptomic changes in response to salinity have been analyzed previously, showing that *CmDOF18* might be associated with resistance to salinity stress in chrysanthemum [8]. Here, we cloned the *CmDOF18* gene from chrysanthemum, and showed that its expression was induced by salinity stress. Transgenic plants were generated to study the function of *CmDOF18*. Overexpression of *CmDOF18* in chrysanthemum caused increased resistance to salinity stress compared with that of the wild-type (WT) plants, while silencing of the gene increased sensitivity to salinity stress. Thus, *CmDOF18* might positively regulate resistance to salinity treatment by regulating the synthesis of oxidoreductase, highlighting a novel chrysanthemum defense mechanism against salinity.

## Materials and methods

### Plant materials and growth conditions

The chrysanthemum cultivar ‘Jinba’ was obtained from the Training Base at the College of Horticulture, Xinyang Agriculture and Forestry University, China. The plants were transplanted into pots filled with a 1:1 (v/v) mixture of soil:vermiculite and grown in a greenhouse under a 16/8 light/dark cycle with a light density of 100 µmol·m^− 2^·s^− 1^ and a relative humidity of 70%. The day/night temperature was 23 °C/18°C.

### Isolation and sequence analysis of CmDOF18

Total RNA was isolated from ‘Jinba’ leaves using RNAiso reagent (TaKaRa, Tokyo, Japan), and then cDNA was obtained using M-MLV reverse transcriptase (TaKaRa, Tokyo, Japan) following the manufacturer’s instructions. A pair of primers (CmDOF18-F/R) was designed to amplify the *CmDOF18* open reading frame by PCR. Gene-specific primers were designed with Primer Premier 5 [32] (Table S1). The PCR products were purified and inserted into the pMD19-T vector for sequencing. The polypeptide sequences of CmDOF18 homologs were selected via BLAST search online (https://www.ncbi.nlm.gov/blast). The amino acid sequences of CmDOF18 and its homologs were aligned using DNAMAN 6.0 software (Lynnon Biosoft; https://www.lynnon.com/). A phylogenetic tree was constructed with MEGA 11 software [33] using the neighbor-joining method, p-distance substitution model and 1,000 bootstrap replicates were selected respectively.

### Expression analysis of CmDOF18 in response to salinity stress and in different tissues

For salinity treatment, plants at the six- to eight-leaf stage were used and watered with 200 mM NaCl [34]. Leaves were sampled at 0, 1, 4, 12, and 24 h after treatment, including three individual biological replicates. The leaves were frozen in liquid nitrogen rapidly, then stored at -80 °C for RNA extraction. The root, stem, leaf, tubular florets, ray florets, and pollen in chrysanthemum were harvested for analysis of *CmDOF18* relative expression in different tissues.

Total RNA from different tissues and leaves of salinity-stressed plants was extracted using RNAiso reagent (TaKaRa, Tokyo, Japan). cDNA was synthesized using M-MLV reverse transcriptase (TaKaRa, Tokyo, Japan). The primer pair CmDOF18-RT-F/R was used for expression of *CmDOF18*, and the *CmEF1α* gene (KF305681) was used as a reference sequence. The expression of *CmDOF18* was detected by quantitative real-time PCR (qRT-PCR) using SYBR® Premix Ex Taq™ II (Tli RNaseH Plus) (TaKaRa, Tokyo, Japan). The resulting data represent three biological replicates. The relative transcript abundances were calculated using the 2^−ΔΔCt^ method.

### Subcellular localization of CmDOF18

To generate the GFP-CmDOF18 fusion construct, the ORF of CmDOF18 was cloned and inserted into the binary vector pMDC43, resulting in the plasmid 35 S::GFP-CmDOF18. Onion (*Allium cepa*) epidermal strips with inner cell surfaces oriented upward were placed on MS medium. 35 S::GFP-CmDOF18 and 35 S::GFP were transiently introduced into onion epidermal cells by a helium-driven particle accelerator (PDS-1000; Bio-Rad, Hercules, CA, USA). Then, the cells were incubated in the dark for 16 h at 22 °C. The GFP fluorescence was examined, and photographs were obtained under a Leica TCS SP8 (Germany) confocal microscope.

### Transcriptional activity analysis

The coding region of *CmDOF18* without a termination codon was cloned and inserted into the pGBKT7 vector with the primer pair CmDOF18-BD-F/R. The constructed plasmid pGBKT7-CmDOF18, pCL1 (positive control) and pGBKT7 (negative control) were transformed into cells of yeast strain Y2H Gold (Clontech, Mountain View, CA, USA) following the manufacturer’s protocol. pCL1 was grown on SD/-Leu medium, while pGBKT7-CmDOF18 or pGBKT7 was plated on SD/-Trp medium. Then, the colonies were transferred to SD/-His-Ade media or SD/-His-Ade media supplemented with adding 40 mg·L^− 1^ X-α-gal and incubated at 30 °C for three days to determine the activation activity.

### Plasmid construction and transformation of chrysanthemum

The *CmDOF18* coding sequence was first cloned by PCR with the primer pair CmDOF18-GATE-F/R, and inserted into the pENTR™ 1 A gateway vector between *Sal* I and *Not* I sites, and then transferred to the overexpression vector pMDC43 by recombinant cloning. To obtain the dominant repressor of *CmDOF18* (*CmDOF18*-SRDX), a 873 bp genomic fragment containing full-length *CmDOF18* open reading frame (ORF) was amplified by PCR and ligated into pENTR™ 1 A vector, and then ligated into p35S-SRDX by recombinant cloning.

The plasmids were introduced into *Agrobacterium tumefaciens* strain EHA105 and then transformed into chrysanthemum using the Agrobacterium-mediated method. Leaf disc (5 mm diameter) from ‘Jinba’ cultured in vitro were used as explants [35]. After regeneration, the expression of *CmDOF18* was tested by qRT-PCR to identify lines with overexpression and silencing. The primer pair CmDOF18-RT-F/R was used to amplify the gene *CmDOF18*, while CmEF1α-F/R was used for the *CmEF1α* gene.

### Salinity treatment of transgenic chrysanthemum

WT and transgenic plants at the 6- to 8-leaf stage were irrigated with 200 mM NaCl for 2 weeks. Then the roots of plants were washed with distilled water, and the plants were repotted in fresh soil (1:1 v/v mixture of soil:vermiculite) to recover for 2 weeks [36]. The survival rate of plants was calculated at this time. The leaves were harvested before salinity treatment (control) and at 48 h after salinity treatment for physiological analysis.

### Analysis of physiological changes in transgenic chrysanthemum

Physiological traits of WT plants, *CmDOF18*-overexpressing plants (43-D18-1, 43-D18-18, 43-D18-21) and *CmDOF18*-SRDX transgenic plants (S-D18-2, S-D18-4, S-D18-6) were measured at 0 and 48 h of salinity treatment. The levels of malondialdehyde (MDA), proline and chlorophyll, the activity of superoxide dismutase (SOD) and peroxidase (POD), and the leaf relative electrolyte conductivity were measured. The levels of MDA and proline were evaluated using an MDA Assay Kit (A003-1-1, Jiancheng, Nanjing, China) by the TBA method and a Proline Assay Kit (A107-1-1, Jiancheng, Nanjing, China) by the colorimetric method. Ethanol extraction was used for chlorophyll determination, and the leaf relative electrolyte conductivity was determined using a P902 conductivity meter (Youke, Shanghai, China). The activity of SOD and POD was assessed using a SOD Assay Kit (A001-3-2, Jiancheng, Nanjing, China) by the WST-1 method and a Peroxidase Assay Kit (A084-3-1, Jiancheng, Nanjing, China) by the colorimetric method following the manufacturer’s instructions.

### Transcriptome analysis of transgenic chrysanthemum

The WT plants, *CmDOF18*-overexpressing plants (43-D18-1, 43-D18-18, 43-D18-21) and *CmDOF18*-SRDX transgenic plants (S-D18-2, S-D18-4, S-D18-6) were used for transcriptome sequencing and analysis (Genome, China). The plants at the six- to eight-leaf stage were watered with 200 mM NaCl, and the leaves of the plants were harvested at 48 h after treatment, including three individual biological replicates. The leaves were frozen in liquid nitrogen rapidly, then stored at -80 °C for RNA extraction. Total RNA was extracted as mentioned above. The integrity and quality of the total RNA were verified using a 2100 Bioanalyzer Nano Kit (Agilent Technologies, Santa Clara, CA, USA). The concentration of RNA was measured with a NanoDrop 2000 spectrophotometer (Thermo Fisher Scientific, Waltham, MA, USA). The total RNA was treated with DNase I (TaKaRa, Tokyo, Japan), and Oligos (dT) were used to isolate the mRNA. The mRNA of each library was sequenced on a DNBSEQ T7 located at the Anoroad Genomics Co., Ltd. (Beijing, China; http://www.genome.cn). The clean data were obtained by removing reads containing adapter, low quality reads and reads with a high content of unknown bases (N) from raw data. The clean reads were mapped by HISAT (v2.1.0) [37] to the chrysanthemum genomic database [38], matched to reference gene sequences by Bowtie2 [39]. Fragments per kilo base per million (FPKM) was used to estimate the expression levels of genes and to compare the differences of gene expression among samples. Blast2GO (v2.5.0) was used to obtain the GO (http://geneontology.org) annotation. The DESeq method was applied to analyze differential gene expression in WT plants, *CmDOF18*-overexpressing plants and *CmDOF18*-SRDX transgenic plants, and the screening threshold was a Q value (adjusted *p* value) < 0.05 and log2foldchange value ≥ 1 or ≤ − 1.

### Statistical analysis

All data were analyzed by one-way analysis of variance using SPSS v17.0 software (SPSS Inc., Chicago, IL, USA). Tukey’s honest significant difference test was employed to identify significantly different trait values.

## Results

### Cloning and sequence analysis of CmDOF18

**Fig. 1:**
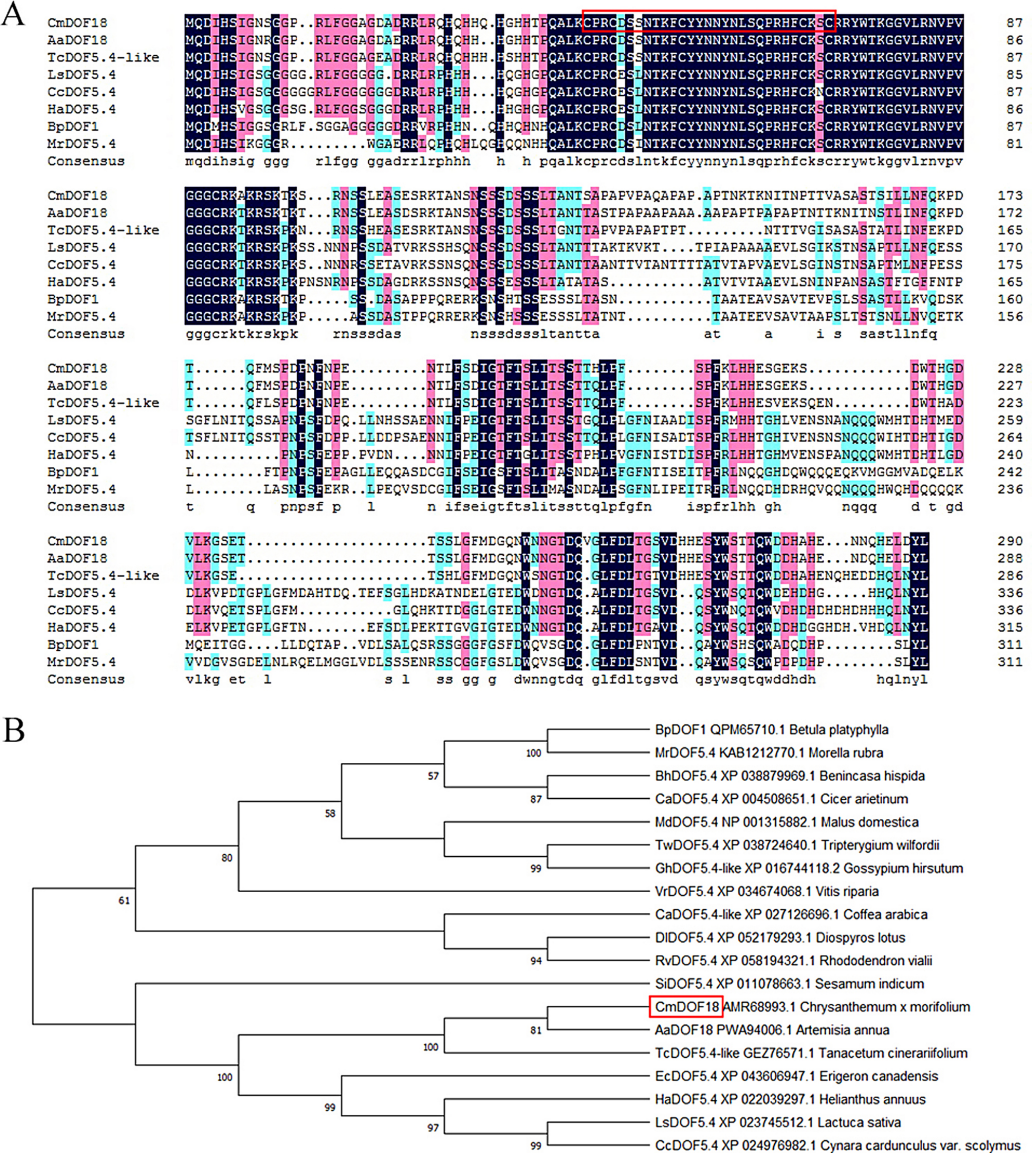
Amino acid sequence of CmDOF18 and phylogenetic tree of CmDOF18. (**A**) Amino acid comparison of CmDOF18 with homologous proteins, while the read box shows Cys2-His2 Zinc Finger domain. (**B**) Phylogenetic analysis of the relationship between CmDOF18 and DOF proteins from other plant species, with the read box showing CmDOF18

The DOF TF *CmDOF18* (KT235692) was isolated from ‘Jinba’ chrysanthemum as described previously [8]. The gene consisted of 1,102 bp with an 873 bp ORF encoding 291 amino acid residues. Amino acid sequence comparisons showed that CmDOF18 contained a typical DOF domain (Fig. 1A). Phylogenetic analysis showed that the sequence of *CmDOF18* was most similar to that of *AaDOD18* from *Artemisia annua* (Fig. 1B).

### Expression profiles of CmDOF18 in response to salinity and in different tissues

**Fig. 2:**
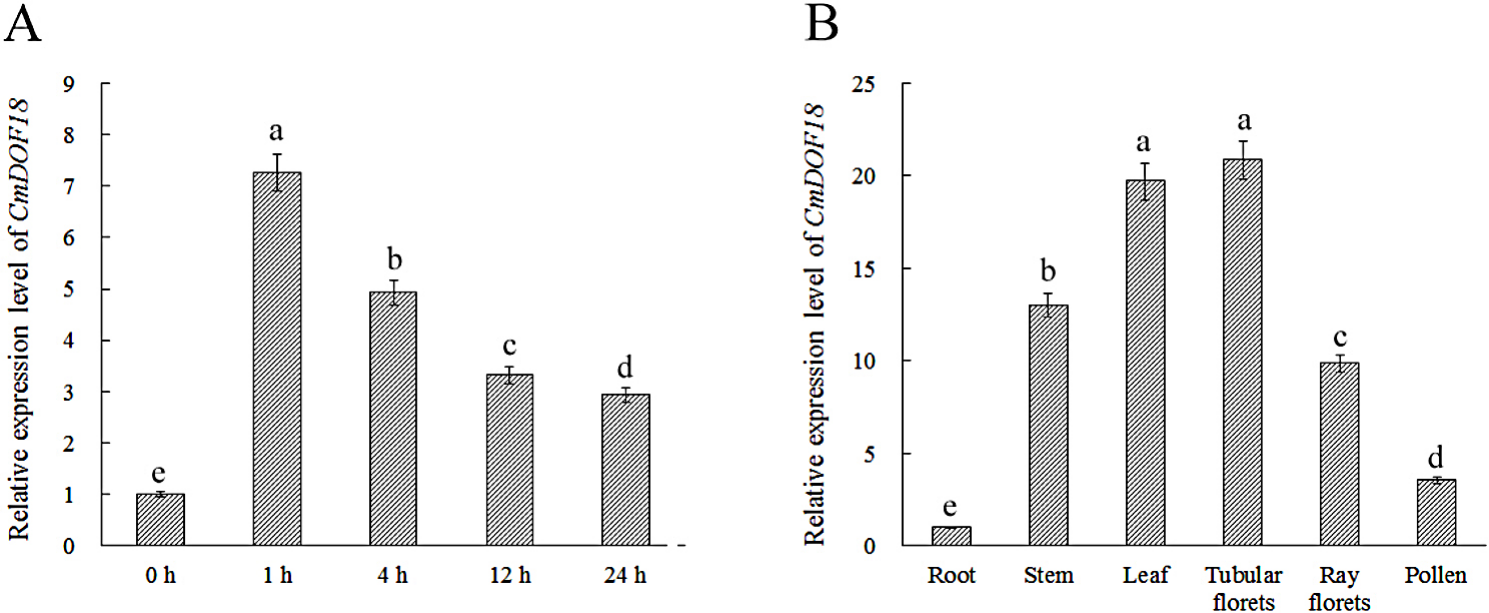
Expression of *CmDOF18* in ‘Jinba’ chrysanthemum under salinity treatment and in different organs as assessed by qRT-PCR. (**A**) Expression patterns of *CmDOF18* in response to 200 mM NaCl treatment. (**B**) Expression of *CmDOF18* in roots, stems, leaves, tubular florets, ray florets and pollen under non-stress conditions

The relative expression levels of *CmDOF18* under salinity treatment were investigated using qRT-PCR. The transcript expression of *CmDOF18* was significantly increased by 7.26-fold at 1 h after salinity stress compared with that in the non-treated plants (Fig. 2A). Thereafter, the relative expression level of *CmDOF18* decreased but was maintained at a higher level than in the untreated control, showing a 2.93-fold increase at 24 h after salinity stress, indicating that *CmDOF18* might regulate the plant’s response to salinity stress.

Transcripts of *CmDOF18* were detected in all tissues analyzed. The results showed that the highest expression level was observed in the tubular florets, followed by those in leaves and stems, while the roots presented the lowest levels of expression (Fig. 2B).

### Subcellular localization and the transcriptional activity of CmDOF18

**Fig. 3:**
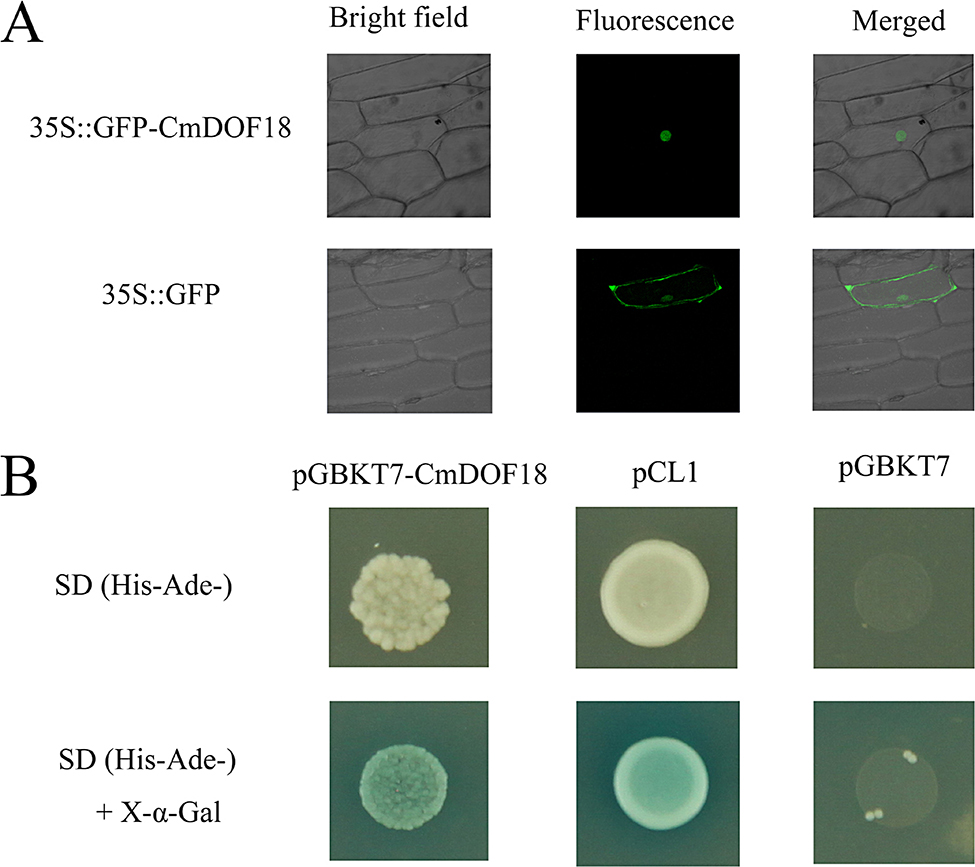
Subcellular localization and transactivation analysis of CmDOF18. (**A**) Subcellular localization of CmDOF18. (**B**) Transformation assay of CmDOF18

The construct 35 S::GFP-CmDOF18 was introduced into onion epidermal cells via particle bombardment. Onion epidermal cells expressing 35 S::GFP-CmDOF18 showed GFP fluorescence localized only in nuclei, while GFP fluorescence localized throughout the onion epidermal cells that was transformed with 35 S::GFP (Fig. 3A). The results indicated that CmDOF18 localized to the nucleus in vivo.

The transcriptional activity of CmDOF18 was measured using yeast one-hybrid expression. The Y2H Gold yeast transformed with pGBKT7-CmDOF18 or pCL1 grew on double-deficient medium, whereas the negative control yeast transformed with pGBKT7 did not grow (Fig. 3B). The results indicated that the whole CmDOF18 protein showed transcriptional activation in yeast cells.

### CmDOF18 contributes to salt resistance of chrysanthemum

**Fig. 4:**
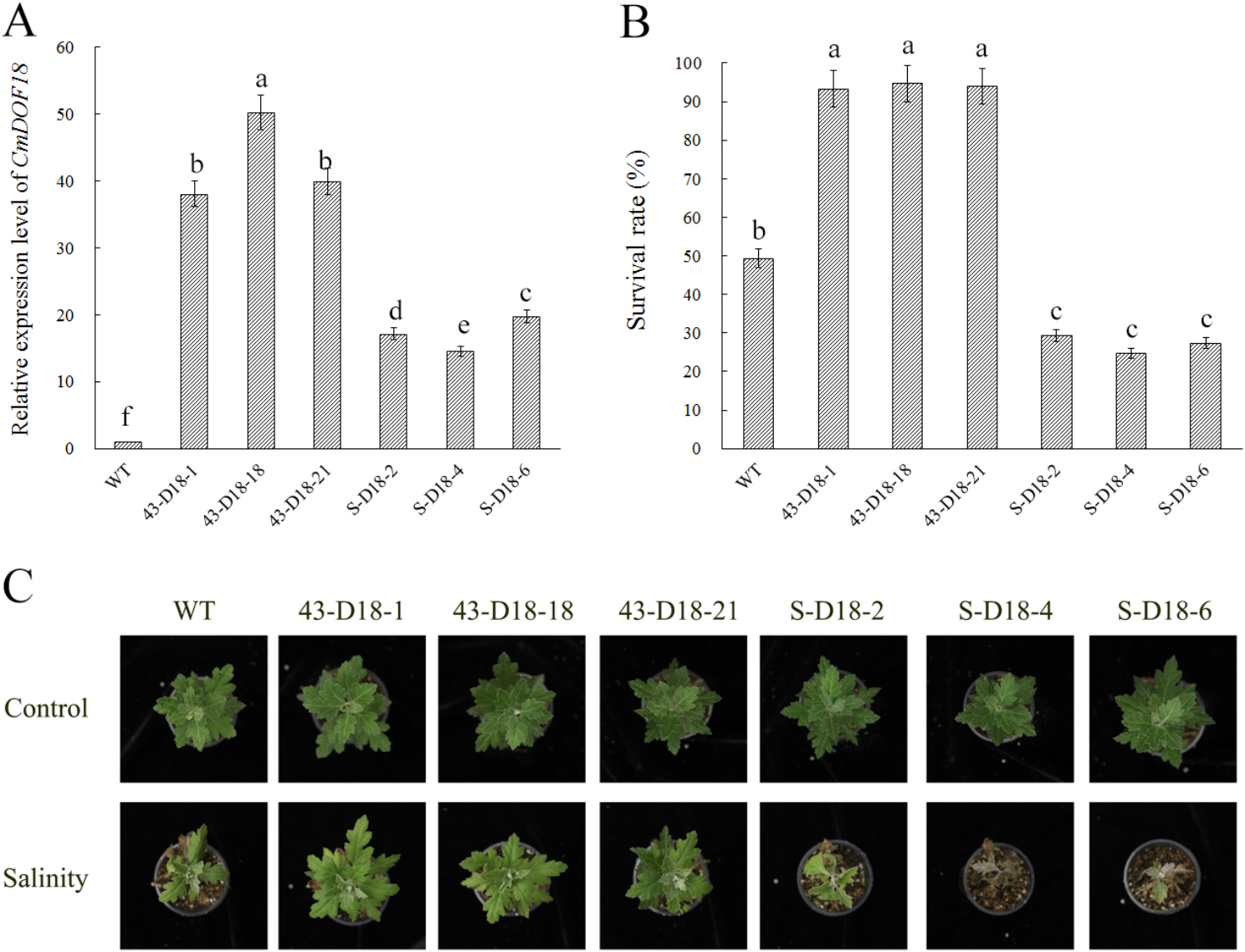
Salinity tolerance of wild-type ‘Jinba’ and transgenic plants. (**A**) Relative expression levels of *CmDOF18* in the transgenic plants. (**B**) Plant survival measured at the end of the recovery period. (**C**) Phenotypic effect of watering with 200 mM NaCl for 2 weeks followed by a 2-week recovery period

To investigate the function of *CmDOF18*, overexpression and gene-silenced transgenic plants were regenerated. The ERF-associated amphiphilic repression (EAR) repression domain (SRDX) is sufficient to convert transcriptional activators into strong repressors, and the dominant repressor acts in a dominant manner in the presence of functionally redundant transcription factors [40–42]. The transcript levels of *CmDOF18* were measured by qRT-PCR (Fig. 4A). The relative expression of *CmDOF18* in overexpressing lines 43-D18-1, 43-D18-18, and 43-D18-21 was significantly increased compared with wild-type plants (WT), and *CmDOF18*-SRDX expression was also significantly increased in S-D18-2, S-D18-4, and S-D18-6, indicating that it significantly suppressed the activity of CmDOF18. *CmDOF18*-overexpressing lines (43-D18-1, 43-D18-18, 43-D18-21) and *CmDOF18*-SRDX gene-silenced lines (S-D18-2, S-D18-4, S-D18-6) were selected for further salinity tolerance evaluation.

After salinity treatment for 2 weeks and recovery for 2 weeks, the survival rates of the transgenic plants were calculated. The percentage survival of *CmDOF18*-overexpressing transgenic plants was 93.33%, 94.67%, and 94.00% for 43-D18-1, 43-D18-18 and 43-D18-21 respectively, which was significantly higher than that of the wild-type plants (49.33%). The survival rate of the dominant inhibited transgenic plants was significantly lower compared with wild-type plants, when the survival rate of *CmDOF18*-SRDX gene-silenced transgenic plants was 29.33%, 24.67% and 27.33% for S-D18-2, S-D18-4, and S-D18-6 respectively (Fig. 4B, C).

**Fig. 5:**
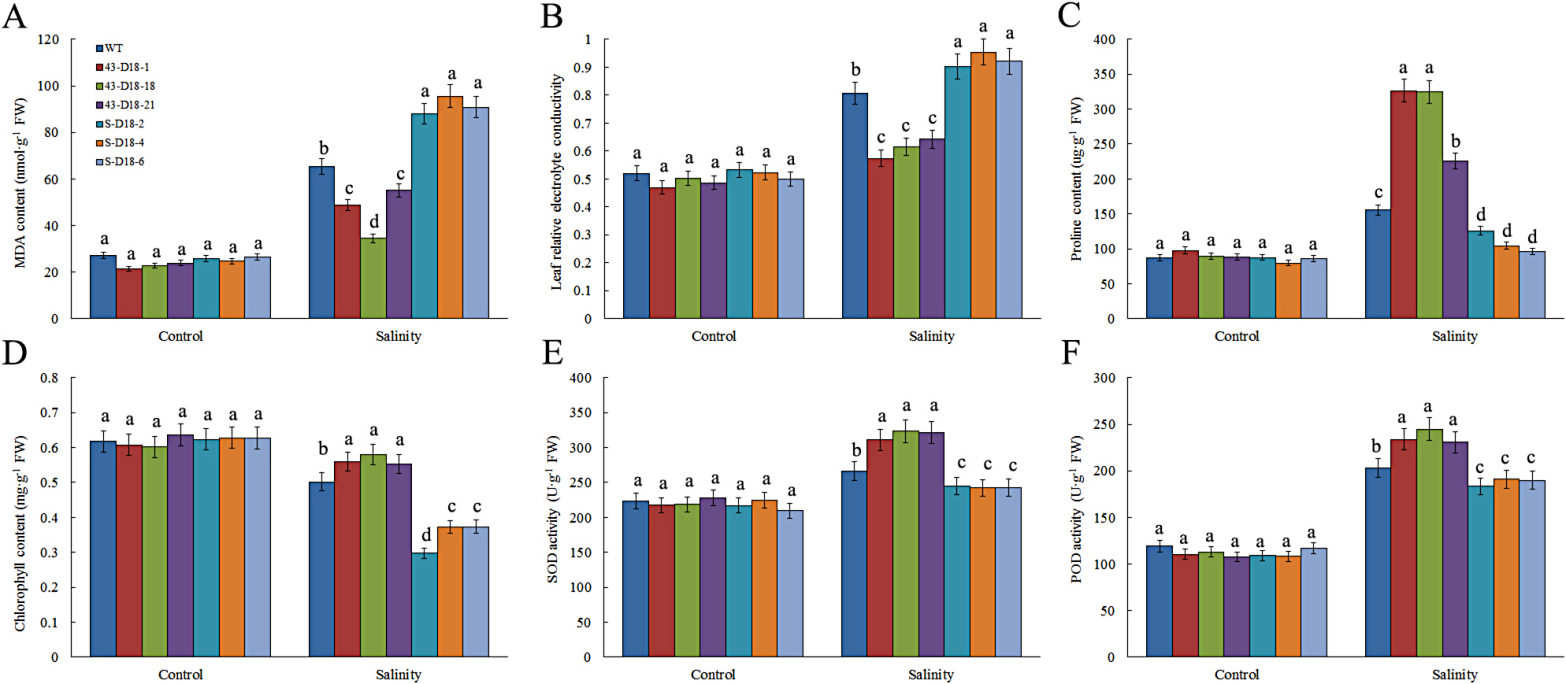
Physiological effects of salinity treatment on wild type ‘Jinba’ and transgenic plants. (**A**) Leaf malondialdehyde (MDA) content. (**B**) Leaf relative electrolyte conductivity. (**C**) Leaf proline content. (**D**) Leaf chlorophyll content. (**E**) Leaf superoxide dismutase (SOD) activity. (**F**) Leaf peroxidase (POD) activity

The leaves of MDA, proline and chlorophyll; the activity of SOD and POD; and the leaf relative electrolyte conductivity were measured in both WT and transgenic plants after 48 h of salinity treatment. There were no differences in MDA, proline, chlorophyll content; electrolyte leakage; or SOD or POD activity in either overexpression or gene-silenced plants compared with WT plants under non-stress conditions. The MDA content and the leaf relative electrolyte conductivity were markedly lower in *CmDOF18*-overexpressing transgenic plants than in WT plants but higher in *CmDOF18*-SRDX gene-silenced transgenic plants than in WT plants (Fig. 5A, B). In contrast, the content of proline and chlorophyll and the activity of SOD and POD were significantly higher in *CmDOF18*-overexpressing transgenic plants than in WT plants, while the numbers were remarkably lower in *CmDOF18*-SRDX gene-silenced transgenic plants than in WT plants (Fig. 5C-F). These results indicated that *CmDOF18* overexpression increased salinity tolerance in chrysanthemum and that *CmDOF18* gene silencing reduced resistance to salinity stress.

### Expression profiles of genes in CmDOF18 transgenic plants

**Fig. 6:**
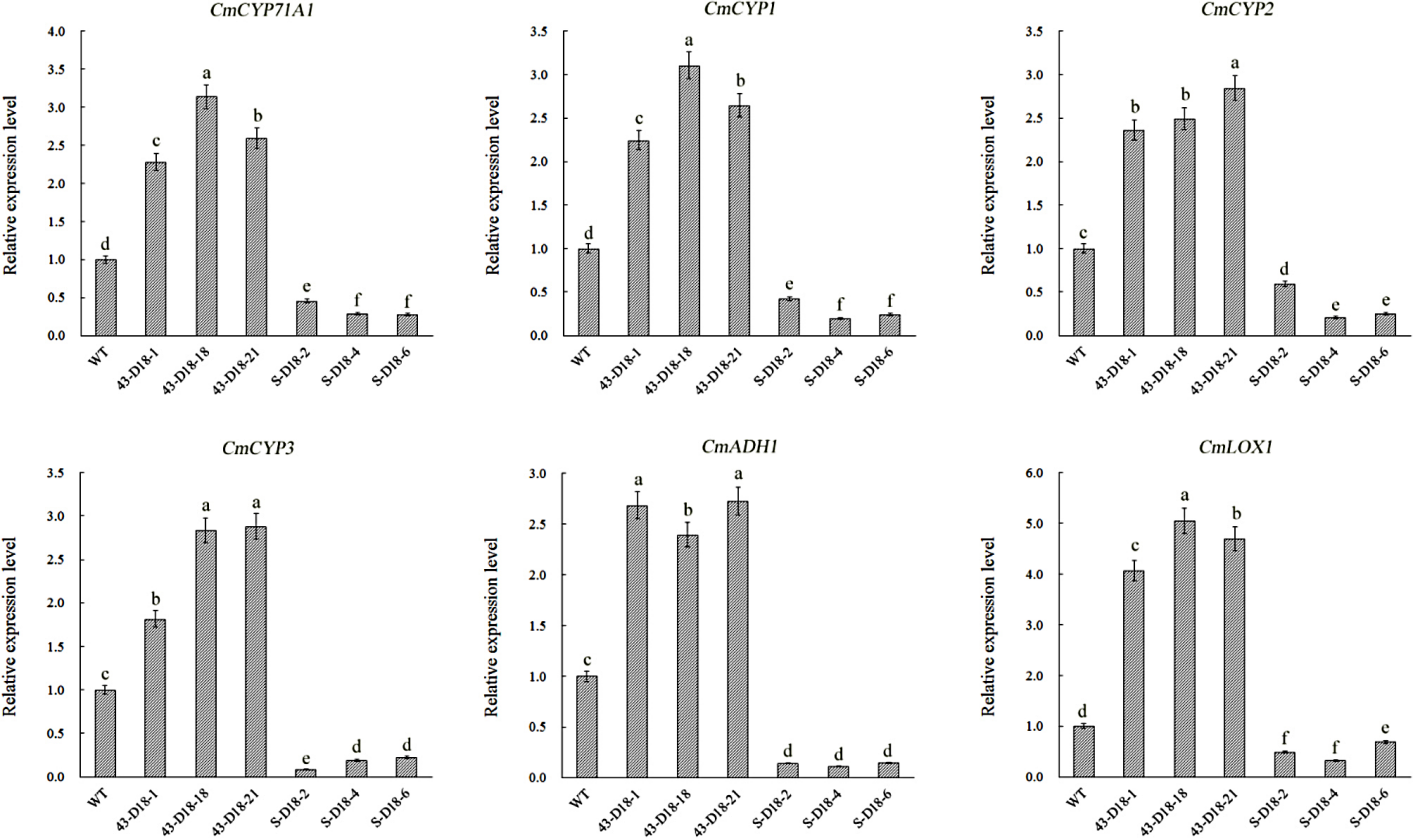
Expression of differentially expressed genes (DEGs) involved in oxidoreductase system of the salinity pathway between wild-type plants and transgenic lines which were treated with 200 mM NaCl for 48 h

The chrysanthemum genome database was used as reference. The raw sequence data were deposited in the NCBI Sequence Read Archive database (Accession number: PRJNA1037609; https://www.ncbi.nlm.nih.gov/sra/PRJNA1037609). Through de novo assembly, 176,913 total clean reads were annotated into chrysanthemum genome database. The differentially expressed genes (DEGs) were identified by comparing wild-type plants versus *CmDOF18-*overexpressing plants (WT vs. OE), wild-type plants versus *CmDOF18-*SRDX gene-silenced plants (WT vs. SRDX), up-regulated in WT vs. OE (WT vs. OE UP), and down-regulated in WT vs. SRDX (WT vs. SRDX DOWN). In the pairwise comparisons of WT vs. OE, and WT vs. SRDX, there were 4,985 (2,375 up-regulated and 2,610 down-regulated), 3,461 (1,974 up-regulated and 1,487 down-regulated) were identified, respectively. The functional enrichment analysis of DEGs in WT vs. OE and WT vs. SRDX revealed 53 (Table S2) and 46 biological process terms (Table S3), respectively, including several terms related to response to stimulus (GO:0050896), catalytic activity (GO:0003824), antioxidant activity (GO:0016209), etc. To further explore the mechanism of *CmDOF18* regulating salt stress resistance, we focused on the DEGs up-regulated in WT vs. OE and down-regulated in WT vs. SRDX. Further analysis showed that 49 GO biological process terms were significantly enriched of the common up-regulated genes in WT vs. OE (Table S4), and 44 GO terms were significantly enriched of the common down-regulated genes in WT vs. SRDX (Table S5). These terms included various processes involved in plant response to abiotic stress, particularly response to stimulus (GO:0050896, 151 DEGs in WT vs. OE UP, 112 DEGs in WT vs. SRDX DOWN), catalytic activity (GO:0003824, 889 DEGs in WT vs. OE UP, 469 DEGs in WT vs. SRDX DOWN ), and antioxidant activity (GO:0016209, 16 DEGs in WT vs. OE UP, 21 DEGs in WT vs. SRDX DOWN). There was an overlap of 25 genes between WT vs. OE UP and WT vs. SRDX DOWN (Fig. S1). Furtherly, 6 oxidoreductase-related genes were found among these overlapping genes, such as *CmCYP71A1* (Chrysanthemum_x_morifolium_newGene_36630), *CmCYP1* (evm.TU.scaffold_11826.43), *CmCYP2* (evm.TU.scaffold_11826.54), *CmCYP3* (evm.TU.scaffold_460.444), *CmADH1* (evm.TU.scaffold_1760.29), and *CmLOX1* (evm.TU.scaffold_7329.167) (Table S6), suggesting that *CmDOF18* might play a role in the plant salinity stress response by modulating the expression of oxidoreductase. The expression levels of the abovementioned DEGs were verified by qRT-PCR (Fig. 6).

## Discussion

### Structural characteristics and transcriptional activation activity of CmDOF18

DOF TFs are characterized mainly by the presence of the conserved DOF DNA-binding domain in the N-terminus and a C-terminal transcriptional activation domain [3, 43, 44]. In this study, *CmDOF18*, a group VI DOF gene, was obtained from chrysanthemum. Sequence analysis showed that it contains a highly conserved DOF domain (Fig. 1A), suggesting that CmDOF18 might be able to bind to the element with the sequence 5’-AAAG-3’. DOF TFs contain a bipartite nuclear localization signal (NLS) that partly overlaps with the conserved DOF DNA-binding domain [45, 46], and the subcellular localization of CmDOF18 showed that it localized to the nucleus. Transactivation assays showed that CmDOF18 is transcriptionally active, suggesting that it might activate the expression of downstream genes to exert its effects.

### CmDOF18 positively regulates the resistance of chrysanthemum to salinity treatment

Several results have previously shown that DOF family members play important roles in resistance to various abiotic stresses in plants [6, 13, 47–51]. *VyDOF8* expression is significantly induced by cold treatment, drought treatment, and salt treatment, and *VyDOF8*-overexpressing tobacco shows enhanced drought tolerance due to increases in abscisic acid and promotion of stress-responsive gene expression [22]. OsDOF15 can bind to the DOF motif in the downstream *OsACS1* promoter and may participate in primary root elongation under salt stress by regulating cell proliferation in the root meristem, via restriction of ethylene biosynthesis [24]. RNA interference (RNAi) of *SlDof22* in transgenic lines increases ascorbic acid (AsA) levels and affects the expression of genes in the D-mannose/L-galactose pathway and AsA recycling, resulting in reduced tolerance to salt stress by significantly downregulating the *SlSOS1* gene [52]. In the present study, *CmDOF18* expression was significantly induced by salinity stress (Fig. 2A), and plants overexpressing *CmDOF18* exhibited improved resistance to salinity stress, while gene-silenced plants showed reduced resistance to salt stress.

Various physiological indices will change under salt stress, such as malondialdehyde, leaf relative conductivity, proline, chlorophyll, SOD, POD, and the degree of these indices can indicate the strength of plant resistance to salt stress [53–55]. MDA, electrolyte leakage, proline, and chlorophyll concentrations are generally used as indicators of plant membrane damage levels under salt stress [31, 56]. As MDA is a product of membrane lipid peroxidation, MDA content can serve as an indicator of the degree of cellular membrane lipid peroxidation occurring as a response to stress [57]. In addition, electrolyte leakage reflects membrane injury caused by stresses [58]. The MDA content and leaf relative electrolyte conductivity in *CmDOF18*-overexpressing lines were lower than those in WT and gene-silenced lines under salt stress, suggesting that *CmDOF18* improved plant salinity tolerance by maintaining the membrane integrity of plants. Proline functions as an osmotic protectant for various cellular structures during episodes of abiotic stress, and its content strongly increases in response to a variety of stresses in plants, such as drought, salt stress, cold injury, etc. [59]. In present study, when the plants suffers from salt stress, the contents of proline showed an increased trend compared with that under non-stress, not only in wild-type plants and *CmDOF18*-overexpressing transgenic plants, but also in *CmDOF18*-SRDX gene-silenced transgenic plants, suggesting that plants could accumulate proline, acting as an osmotic regulator, to regulate the response to salt stress, which is entirely consistent with previous reports [60]. Further analysis showed that more proline accumulated in the leaves of *CmDOF18*-overexpressing lines than that in WT, lower proline accumulated in gene-silenced lines compared with that in WT under salt stress, suggesting that *CmDOF18* might improve plant salinity tolerance potentially by accumulation of proline. Chlorophyllase activity increases under salt stress, leading to decreased chlorophyll content [61]. In our study, we found that the content of chlorophyll decrease in plants under salt stress, and higher content in *CmDOF18*-overexpressing lines, lower content in gene-silenced lines than that in wild-type plants, meaning that *CmDOF18* might response to salt stress by regulating synthesis or degradation of chlorophyll. Oxidative damage is caused by the accumulation of reactive oxygen species (ROS), which occurs under various stresses in plants [30]. The two enzymes SOD and POD are involved in oxidative protection [62]. SOD catalyzes O^2−^ to produce oxygen and H_2_O_2_ by catalyzing the dismutation reaction, and POD metabolizes H_2_O_2_ to H_2_O through synergistic action. Consistent with this result, the SOD and POD activity under salinity stress was higher in *CmDOF18*-overexpressing lines than in WT and gene-silenced lines, suggesting that these enzymes contribute to improving the resistance to salinity stress in *CmDOF18*-overexpressing lines. These results suggested that *CmDOF18* genes might resist salinity stress by regulating lipid peroxidation, osmoregulatory substance, and ROS accumulation in plants.

### CmDOF18-altered salt resistance is potentially related to oxidoreductase

DOF proteins can respond to salt stress by regulating a variety of pathways, including osmotic substance synthesis, protective enzyme synthesis, Na + excretion, and so on [63, 64]. ThDof1.4 could increase the proline level and enhance ROS scavenging capability to improve salt and osmotic stress tolerance in *Tamarix hispida* [48]. TaZNF, a wheat DOF protein, significantly improved salt tolerance by controlling the expression of many downstream genes to increase Na + excretion in Arabidopsis [49]. Here, transcriptome analysis showed that the identified differently expressed genes between wild-type and CmDOF18 transgenic plants are mainly involved in oxidoreductase activity (Table S6). Oxidoreductase is an enzyme that catalyzes oxidation-redution reactions, which exist widely in organisms. The main function of oxidoreductase is to produce energy and synthesize various substances needed for plant growth and the interaction between plants and the environment. Cytochromes P450s (P450s) are a large superfamily of heme-containing monooxygenases, that function in metabolic detoxification and participate primarily in the synthesis of plant secondary metabolites and in plant defense [65]. The expression of *PtCYP714A3*, a cytochrome P450 monooxygenase gene, is greatly induced by salt and osmotic stress in plants, and transgenic rice plants exhibit enhanced tolerance to salt and maintained more Na^+^ in both shoot and root tissues under salinity stress than WT plants, suggesting that *PtCYP714A3* plays a crucial role in shoot responses to salt toxicity in rice by regulating gibberellin synthesis [66]. Alcohol dehydrogenases (ADHs) in plants are encoded by a multigene family, which participates in growth, development, and adaptation in many plant species. *ScADH3*, which maintains the steady state of ROS by regulating ROS-related genes, is also related to cold tolerance in transgenic tobacco, as indicated by functional analysis [67]. In plants, lipoxygenases (LOXs) are involved in various physiological processes, including defense responses to biotic and abiotic stresses. *CaLOX1* plays a crucial role in plant stress responses by modulating ABA- and stress-responsive marker gene expressions, lipid peroxidation and H_2_O_2_ production [68]. *CmLOX10* positively regulates drought tolerance through jasmonic acid -mediated stomatal closure in oriental melon [69]. In the present study, we found that the expression of six oxidoreductase genes, including *CmCYP71A1*, *CmCYP1*, *CmCYP2* and *CmCYP3* encoding cytochrome P540 monooxygenase; *CmADH1* encoding an alcohol dehydrogenase; and *CmLOX1* encoding a lipoxygenase, was increased in *CmDOF18*-overexpressing plants but decreased in SRDX lines. Thus, we propose that *CmDOF18* mediates resistance to salinity stress and that the mechanism could be related to oxidoreductases such as cytochrome P450 monooxygenases, alcohol dehydrogenases, and lipoxygenases. However, the specific mechanism remains to be clarified, and more data are needed before a definitive conclusion can be made.

**Fig. 7:**
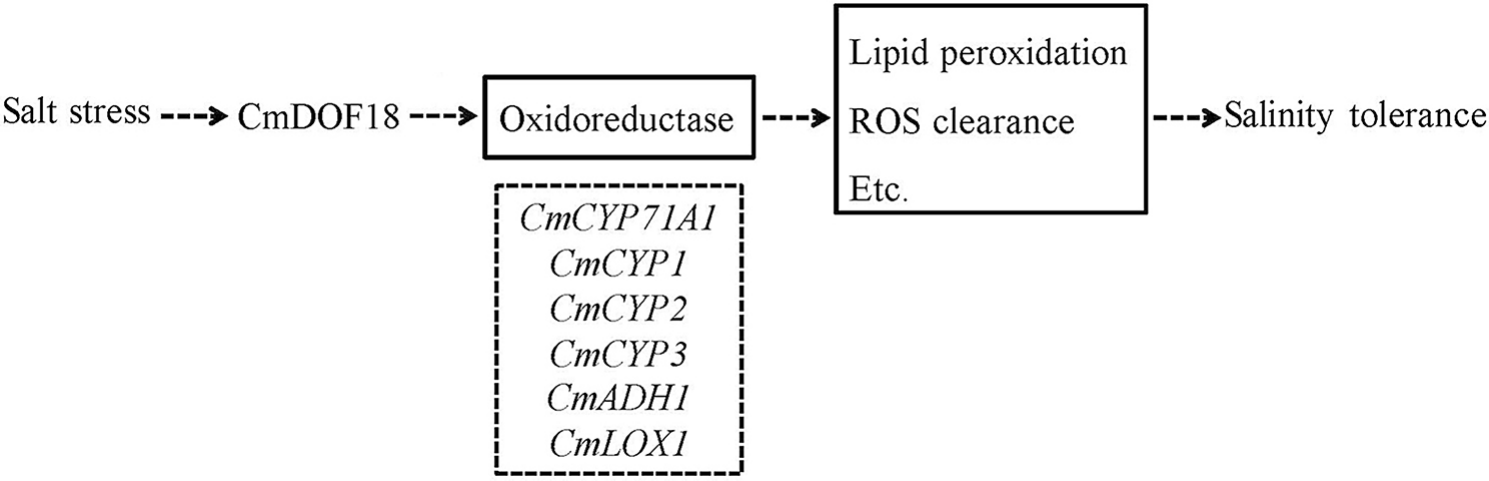
Hypothetical model for CmDOF18 function during salt stress. Accumulation of CmDOF18 during salinity stress results in an increase in oxidoreductase gene expression. Genes shown dotted box are those oxidoreductase genes (*CmCYP71A1*, *CmCYP1*, *CmCYP2*, *CmCYP3*, *CmADH1*, and *CmLOX1*) that responded to salt stress

## Conclusion

In summary, *CmDOF18* was cloned from chrysanthemum, and its expression was induced by salinity stress, indicating that *CmDOF18* mediates resistance to salinity stress in chrysanthemum. The expression levels of oxidoreductase genes (*CmCYP71A1*, *CmCYP1*, *CmCYP2*, *CmCYP3*, *CmADH1*, and *CmLOX1*) increased in *CmDOF18*-overexpressing plants but decreased in *CmDOF18*-SRDX gene-silenced plants. It appeared that CmDOF18 activates the above genes in *CmDOF18*-overexpressing lines during salt stress, which therefore results in tolerance to salinity (Fig. 7).

### Electronic supplementary material

Below is the link to the electronic supplementary material.

Supplementary Material 1

Supplementary Material 2

Supplementary Material 3

Supplementary Material 4

Supplementary Material 5

Supplementary Material 6

Supplementary Material 7

## Data Availability

The raw RNAseq data has been successfully uploaded to NCBI and the accession number for our submission is: PRJNA1037609. The materials used during the current study are available from the corresponding author on reasonable request.

## Abbreviations

DOF: DNA binding with one finger
TFs: Transcription factors
ORF: Open reading frame
MDA: Malondialdehyde
SOD: Superoxide dismutase
POD: Peroxidase
FPKM: Fragments per kilo base per million
WT: Wild-type
EAR: ERF-associated amphiphilic repression
DEGs: Differentially expressed genes
RNAi: RNA interference
ROS: Reactive oxygen species
P450s: Cytochromes P450s
ADHs: Alcohol dehydrogenases
LOXs: Lipoxygenases
