# Supplementary material for: CmDOF18 positively regulates salinity tolerance in Chrysanthemum morifolium by activating the oxidoreductase system
Source: BMC Plant Biol. 2024 Apr 1;24:232. doi: 10.1186/s12870-024-04914-y (PMC10985857; doi:10.1186/s12870-024-04914-y)
Supplement: Supplementary file 1 — Supplementary Material 1 [file 12870_2024_4914_MOESM1_ESM.docx]

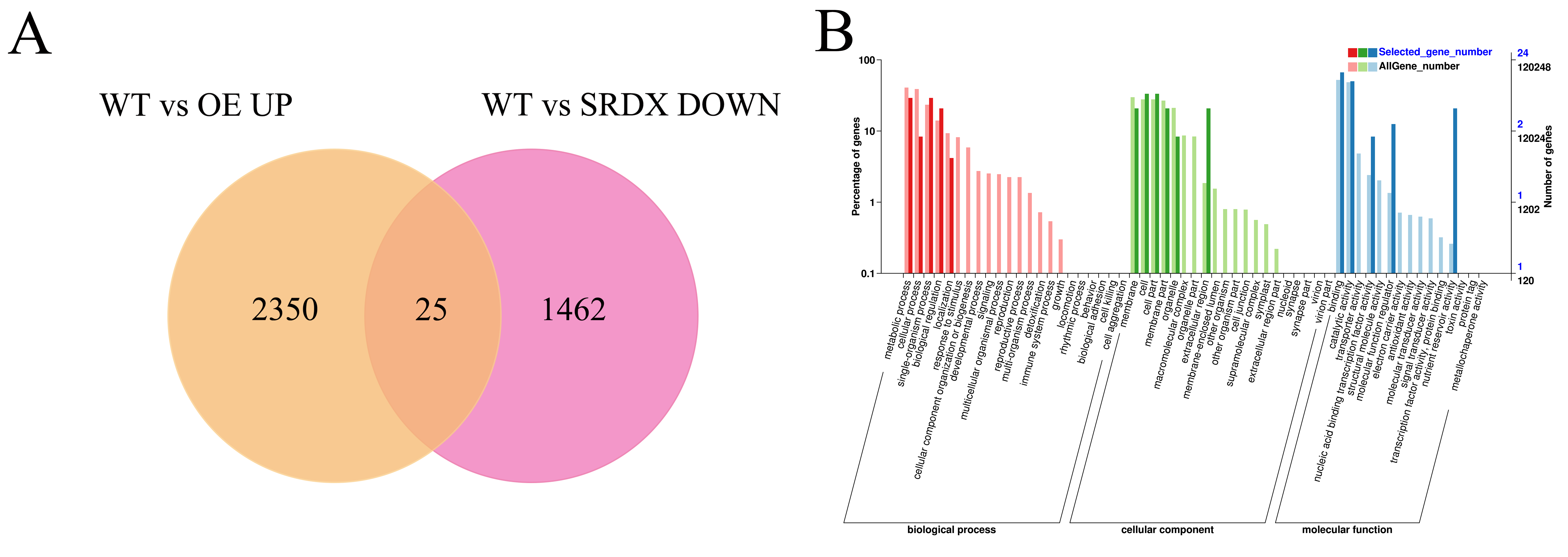


**Figure S1** The RNA-seq analysis of WT and *CmDOF18* transgenic lines. **A** Venn diagram of DEGs of the comparison between WT and transgenic plants. **B** Gene Ontology (GO) functional classification of allgenes (the total genes which were annotated into chrysanthemum genome database) and selected genes (25 overlapping DEGs).
